# Body Contouring Using a Combination of Pulsed Ultrasound and Unipolar Radio Frequency: A Prospective Pilot Study

**DOI:** 10.1007/s00266-022-02919-2

**Published:** 2022-06-01

**Authors:** Fernando Urdiales-Gálvez, Sandra Martín-Sánchez, Mónica Maíz-Jiménez, Esther Viruel-Ortega

**Affiliations:** Instituto Médico Miramar, P. Miramar, 21, 29016 Malaga, Spain

**Keywords:** Body contouring, Radiofrequency, Non focus pulsed ultrasound, Fat thickness reduction, Adipose tissue reduction

## Abstract

**Objective:**

To assess the efficacy and safety of a new non-invasive body contouring device in patients with localized fat in abdomen or in abdomen and hips. Additionally, we also evaluated the patient satisfaction with the procedure.

**Methods:**

Prospective and non-randomized open label study. The patients underwent four sessions, separated by 1 week each, with the Alma PrimeX, a non-invasive body contouring device that combines pulsed non-focus ultrasound and a Unipolar radiofrequency. The primary end point was the mean change in fat tissue thickness, assessed by diagnostic ultrasound, from baseline to 3-months after the last treatment-session.

**Results:**

Fifteen subjects were evaluated. As compared to pre-treatment thickness, Hodges-Lehmann median difference (95% CI) was − 85.3 (− 107.5 to − 62.0) mm, *p* = 0.0001; − 70.3 (− 95.0 to − 48.5) mm, *p* = 0.0001; − 100.0 (− 140.5 to − 49.5) mm, *p* = 0.0039; and − 71.8 (− 132.5 to − 23.0) mm, *p* = 0.0078 in infraumbilical, supraumbilical, right hip, and left hip, respectively. Pretreatment fat volume was significantly reduced from 32.9% to 31.2%, *p* = 0.0006. The median (interquartile range) degree of patient satisfaction was 4.0 (1.0–5.0), with 13 (86.7%) patients being “Highly satisfied” or “Satisfied” with the treatment results. The most common adverse event was discomfort, followed by erythema. All the adverse events were mild and were successfully resolved without treatment.

**Conclusions:**

Combine therapy of a Pulsed non-focus ultrasound and Unipolar radiofrequency using the non-invasive device Alma PrimeX was an effective and safe treatment for reducing fat tissue thickness in abdomen and hips in patients with localized fat. Patients’ satisfaction with the procedure was high.

**Level of Evidence IV:**

This journal requires that authors assign a level of evidence to each article. For a full description of these Evidence-Based Medicine ratings, please refer to the Table of Contents or the online Instructions to Authors www.springer.com/00266.

## Introduction

There is an increasing pressure for looking good and healthy in today’s society. This is not only due to the ubiquitous of idealized bodies in the media, but also to a better knowledge of the detrimental effects of obesity [1–4].

Most of the surgical body contouring methods have been associated with several complications, including pain, swelling, prolonged recovery, scarring, hematoma or infection [5, 6]. These issues have led to the development of non-invasive techniques. Non-invasive body contouring has experienced a significant and rapid increased in recent years, mainly due to their favorable safety profile, minimal recovery time and reduced cost, while maintained a good efficacy profile [1–5, 7].

The main goal of non-invasive contouring devices is to improve body’s appearance by removing the excess adipose tissue, particularly in areas in which fat persists despite optimal diet and exercise routine [1–5].

Four main non-invasive techniques, for reducing localized subcutaneous adipose tissue, have emerged as preferred options: low-level laser therapy, cryolipolysis, radio frequency (RF) and high-intensity ultrasound, non-focused or focus (HIFU) [1–5]. However, up to date, no procedure has been accepted as the “gold standard”.

Radiofrequency devices are currently the most popular noninvasive body contouring devices used in practice. Although initially used for treating periorbital wrinkles, rhytids, and skin laxity [8, 9]; RF is now widely used for body contouring, skin tightening, and cellulite reduction [10]. This method is based on the difference in water and impedance between skin (low impedance) and fat tissue (high impedance) [3]. RF induces thermal injury to target tissue layers by focusing thermal energy on high impedance tissues, which induces apoptosis of subcutaneous adipose tissue cells with minimal risk of damaging other tissues, such as epidermis or muscle [11, 12].

High-intensity focused and non-focused ultrasounds had been introduced as a non-invasive body contouring strategy that focus mainly on skin tightening and rejuvenation [3]. It uses acoustic energy to induce fat tissue apoptosis. HIFU devices can deliver energy to the deep dermis, subdermal connective tissue, and fibromuscular layers in precise zones without damage to the epidermis. Besides its thermal effects, HIFU also has a mechanical effect that causes an immediate disruption of cell membranes, which favor apoptosis and necrosis [3]. Regarding non-focused ultrasound, its effect on adipose tissue seems to be related to apoptosis and cavitation, which causes a mechanical disruption of subcutaneous adipocytes [13, 14].

HIFU effects are easily perceived since there is a clear border between targeted and untargeted tissue [15]. Moreover, HIFU is thought to cause gradual skin tightening through collagen contraction and remodeling [16].

Non-focused ultrasound and electrical stimulation are therapeutic modalities commonly used in physiotherapy practice. In fact, the combination of electrical stimulation and ultrasound can be more effective than each of them used separately [17].

Alma PrimeX (Alma Lasers, Ltd. Caesarea, Israel) is a non-invasive body contouring platform that combines a unique pulsed non-focus ultrasound and unipolar radiofrequency.

Literature review revealed no evidence of previous attempt of evaluating the effect of the combination of pulsed non-focus ultrasound and unipolar radiofrequency on fat tissue reduction.

The purpose of the current study is to assess the efficacy and safety of this new non-invasive body contouring platform in patients with localized fat in abdomen or in abdomen and flanks. Additionally, we also evaluated the patient satisfaction with the procedure.

## Methods

### Design

This was a prospective and non-randomized open label study. The study protocol was approved by the Ethics Committee of the Instituto Médico Miramar (Málaga, Spain). Written informed consent was provided by all the participants before treatment course. The study protocol adhered to the tenets of the Declaration of Helsinki and the Good Clinical Practice/International Council for Harmonization Guidelines.

### Patients

Patients with localized fat in abdomen or in abdomen and flanks were candidates for undergoing treatment for fat reduction. Eligible participants were healthy women or men aged ≥ 30 years who had clearly visible fat deposits on their abdominal region or abdominal and hip regions with a body mass index (BMI) < 30 kg/m^2^; proportion of fat mass < 25% (women) and < 42% (men), visceral fat < 9, or fat tissue thickness in abdominal area higher than 0.5 cm measured using a composition analyzer.

Patients were excluded if they had undergone previous surgical or non-surgical fat reduction procedures in the area of treatment; have a BMI > 30 kg/m^2^; have implanted electronic devices (pacemakers, insulin pumps, etc.); have presence of metal prostheses in the area to be treated; any inflammatory dermatological diseases (psoriasis, lichen planus, etc.); or have any condition that prevents or recommends not doing this treatment.

### Assessments

Before treatment, all the patients underwent a quantitative and qualitative evaluation of their fat distribution, according to their individual metameric distribution.

The amount and distribution of fat was analyzed macroscopically by means 2D photos with SonyDSC-HX400V (Sony Group Corporation Konan Minato-ku, Tokyo, 108-0075 Japan) and 3D photographs with Canfield Vectra H2 (Canfield Scientific Inc; Parsippany-Troy Hills, NJ 07054, USA). Bioelectrical Impedance Analysis was performed with Tanita BF-180 (Tanita Corporation; Itabashi-Ku, Tokyo, Japan 174-8630). Fat thickness was assessed by ultrasound technology with the Samsung HS 30 ultrasound machine (Samsung Healthcare Global, Gangwon; South Korea).

### Treatment Procedure

The treatment has been performed with the Alma PrimeX (Alma Lasers, Ltd. Caesarea, Israel) non-invasive body contouring device. Its main technical characteristics are summarized in Table 1.

**Table 1:** Main technical characteristics of the non-invasive body contouring device.

Radiofrequency (UniBody applicator)^a^	Ultrasound (UlatraWave applicator)^b^
∙ Power: Up to 300 W	∙ Ultrasonic intensity: Up to 3 W/cm^2^
∙ Operating frequency: 40.68 MHz	∙ Operating frequency: 64 ± 5% kHz continuous/modulated output
∙ Cooling: thermoelectric cooling	∙ Vibration frequency: 0, 10, 20 Hz
∙ Vacuum pump: Up to 0.045 MPa	∙ Control: adaptative digital frequency control
	∙ Cooling: water cooling, air cooling

Patients received a total of four sessions separated by 1 week each

^a^Radiofrequency was applied only in the abdominal region in a 15 min session with an accumulative energy of 70 kJ

^b^Ultrasound was applied in both abdominal and hip regions with a power level 8 *max intensity) and operating frequencies of 10 Hz, with high vacuum, during a session of 15 min

Ultrasound was administered in both abdominal and hip regions with a power level that ranged from 5 to 8 W and operating frequency of 10 Hz, with high vacuum, during a session of 15 min. RF treatment was administered, only in the abdominal region, in a 15-minute session with an accumulative energy of 70 kJ and a power that ranged from 140 to 200 W in sub-deep or deep planes.

Patients received a total of four sessions separated by 1 week each.

Treatment technique with the ultrasound involved an in-motion circular fashion in which according to the size of the treated abdomen would have been treated as a whole or divided into two equal segments, each treated for 7.5 mins. Using the radiofrequency an in-motion circular or brushing linear strokes where performed.

### Patient Satisfaction

A five-point Likert scale (1 = highly dissatisfied; 2 = dissatisfied; 3 = neutral; 4 = satisfied; and 5 = highly satisfied) was used to assess the degree of patient satisfaction with the treatment results by referring to the following question: [1] How satisfied are you with the treatment results?

### Outcomes

The primary outcome was the mean fat thickness reduction in abdominal and hip regions, assessed by diagnostic ultrasound, from pretreatment values to 3 months after the last treatment session.

Secondary outcomes include results of patient satisfaction survey, assessed 3 months after the last treatment session and safety.

### Statistical Analysis

Statistical analysis was performed with MedCalc Statistical Software version 20.015 (MedCalc Software Ltd, Ostend, Belgium; https://www.medcalc.org; 2021).

Continuous variables were presented as median and 95% confidence interval (95% CI) or median (Interquartile range, IqR), as needed; while categorical variables were presented as number (percentage).

The comparisons between pretreatment and posttreatment procedure measurements were performed using the Wilcoxon test, including the Hodges-Lehmann median difference and its 95% CI.

The Mann–Whitney *U* test was used to compare pretreatment values between subjects who underwent treatment in abdomen versus those who underwent treatment in abdomen and hip.

Analysis of covariance (ANCOVA) was used to assess the changes in Infraumbilical and supraumbilical thickness between subjects who underwent treatment in abdomen and those who did it in abdomen and hip. The model included “treated area” as factor and age, weight, BMI, visceral fat, fat mass, pretreatment infraumbilical and supraumbilical thickness as covariates.

## Results

### Pretreatment Demographic and Clinical Characteristics

Twenty subjects, 16 (80.0%) women and 4 (20.0%), were included in the study. Five of these did not attend follow-up visits due to the government-enacted lockdowns as a consequence of coronavirus disease 2019 (COVID-19) outbreak.

A total of 15 patients, 13 (86.7%) women, of the 20 treated attended follow-up visits. Median power of RF (IqR) was 170.0 (155.0–170.0) W administered in sub-deep (5/15 patients) or deep (10/15 patients) planes, with no differences between them (Hodges-Lehmann median difference = 20.0 W; 95% CI − 20.0–30.0 W, *p* = 0.2046).

The mean age was 43.5 ± 9.0 years. Six (40.0%) patients underwent treatment in abdomen and 9 (60.0%) in abdomen and hips.

Table 2 summarizes the main pretreatment parameters in the overall study sample. There were no significant differences between those subjects who underwent treatment in the abdomen or in abdomen and hips in any of the study variables.

**Table 2:** Overview of the main clinical and demographic characteristics of the study sample.

	Overall (*n* = 15)	Abdomen (*n* = 6)	Abdomen & hips (*n* = 9)	*p*^a^
Age, years				0.9059
Mean (SD)	43.5 (9.0)	43.7 (10.5)	43.3 (8.6)	
Median (IqR)	41.0 (35.0–51.5)	44.0 (34.0–50.0)	41.0 (35.0–52.5)	
Sex, *n* (%)				0.4857^b^
Woman	13 (86.7)	6 (100.0)	7 (77.8)	
Man	2 (13.3)	0 (0.0)	2 (22.2)	
Weight, kg				0.3458
Mean (SD)	68.4 (12.5)	63.7 (9.1)	71.5 (14.0)	
Median (IqR)	66.7 (59.0–75.3)	62.9 (55.4–71.6)	69.3 (61.1–82.3)	
BMI, kg/m^2^				0.4094
Mean (SD)	24.8 (3.9)	23.9 (3.3)	25.4 (4.3)	
Median (IqR)	24.4 (22.1–27.1)	22.8 (21.9–27.6)	25.1 (22.7–26.7)	
Visceral fat				0.4383
Mean (SD)	5.5 (2.6)	4.7 (3.0)	6.0 (2.4)	
Median (IqR)	5.0 (3.3–7.8)	4.0 (2.0–8.0)	5.0 (4.8–7.3)	
Fat mass, %				0.8132
Mean (SD)	31.0 (6.9)	31.5 (6.7)	30.7 (7.4)	
Median (IqR)	32.9 (26.9–36.2)	32.7 (26.4–36.2)	32.9 (26.6–35.3)	
FTIU, mm				0.4094
Mean (SD)	216.4 (86.5)	228.3 (65.1)	208.4 (101.4)	
Median (IqR)	195.0 (154.3–275.0)	220.0 (183.0–256.0)	182.0 (128.0–280.5)	
FTSU, mm				0.9062
Mean (SD)	167.4 (80.5)	180.8 (88.9)	158.4 (78.8)	
Median (IqR)	174.0 (105.0–217.0)	168.5 (119.0–176.0)	179.0 (81.0–230.3)	
FTRH				N.A.
Mean (SD)	195.6 (100.9)	N.A.	195.6 (100.9)	
Median (IqR)	189.0 (112.8–289.3)		189.0 (112.8–289.3)	
FTLH^c^				N.A.
Mean (SD)	178.6 (95.7)	N.A.	178.6 (9.5.7)	
Median (IqR)	182.0 (90.5–264.5)		182.0 (90.5–264.5)	

*SD* Standard deviation, *IqR* Interquartile range, *BMI* Body mass index, *FTIU* Fat thickness infraumbilical region, *FTSU* Fat thickness supraumbilical region, *FTRH* Fat thickness right hip, *FTLH* Fat thickness left hip, *N.A.* Not applicable.

^a^Mann-Whitney *U* test

^b^Fisher exact test

^c^Eight subjects

### Fat Thickness Reduction

As compared to pre-treatment values, fat thickness in the infraumbilical region was significantly reduced from 195.0 mm (154.3–275.0 mm) to 118.0 mm (6.0–155.0 mm), *p* = 0.0001. Pre-treatment fat thickness in the supraumbilical region was significantly reduced from 174.0 mm (95% CI 105.0–217.0 mm) to 81.0 mm (95% CI 56.8–123.5 mm), *p* = 0.0001. Similarly, pre-treatment fat thickness in the right hip was significantly reduced from 189.0 mm (95% CI 112.8–289.3 mm) to 69.0 mm (95% CI 51.0–115.3 mm), *p* = 0.0039; while pre-treatment fat thickness in the left hip reduced from 182.0 mm (95% CI 90.5–264.5 mm) to 69.0 mm (63.5–139.0 mm), *p* = 0.0078.

**Fig. 1:**
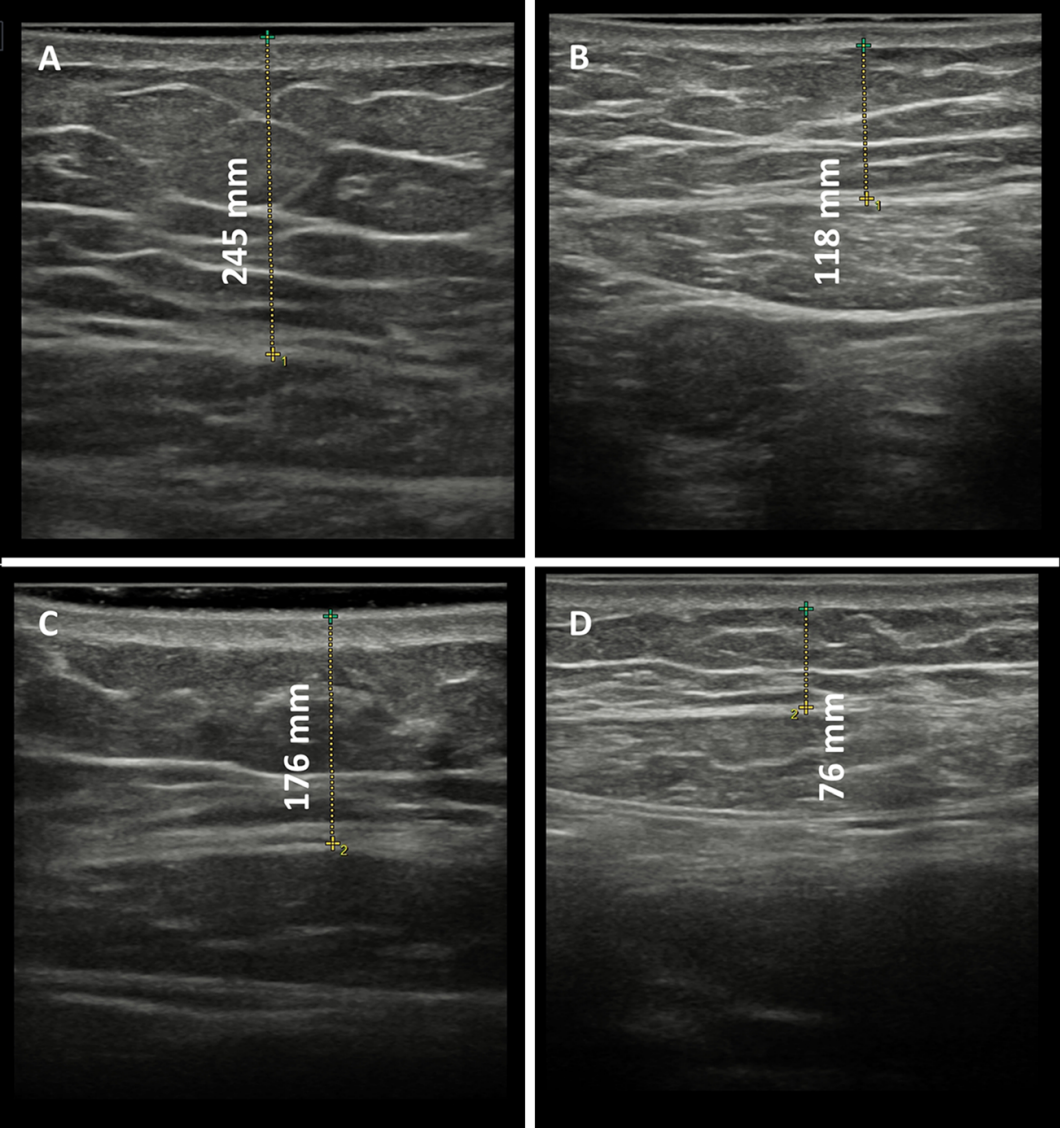
Ultrasound imaging of a 47-year-old woman who underwent four sessions in abdomen with the new non-invasive body contouring device Accent Prime X. **A** Infraumbilical thickness before treatment. **B** Infraumbilical thickness after treatment. **C** Supraumbilical thickness before treatment. **D** Supraumbilical thickness after treatment

**Fig. 2:**
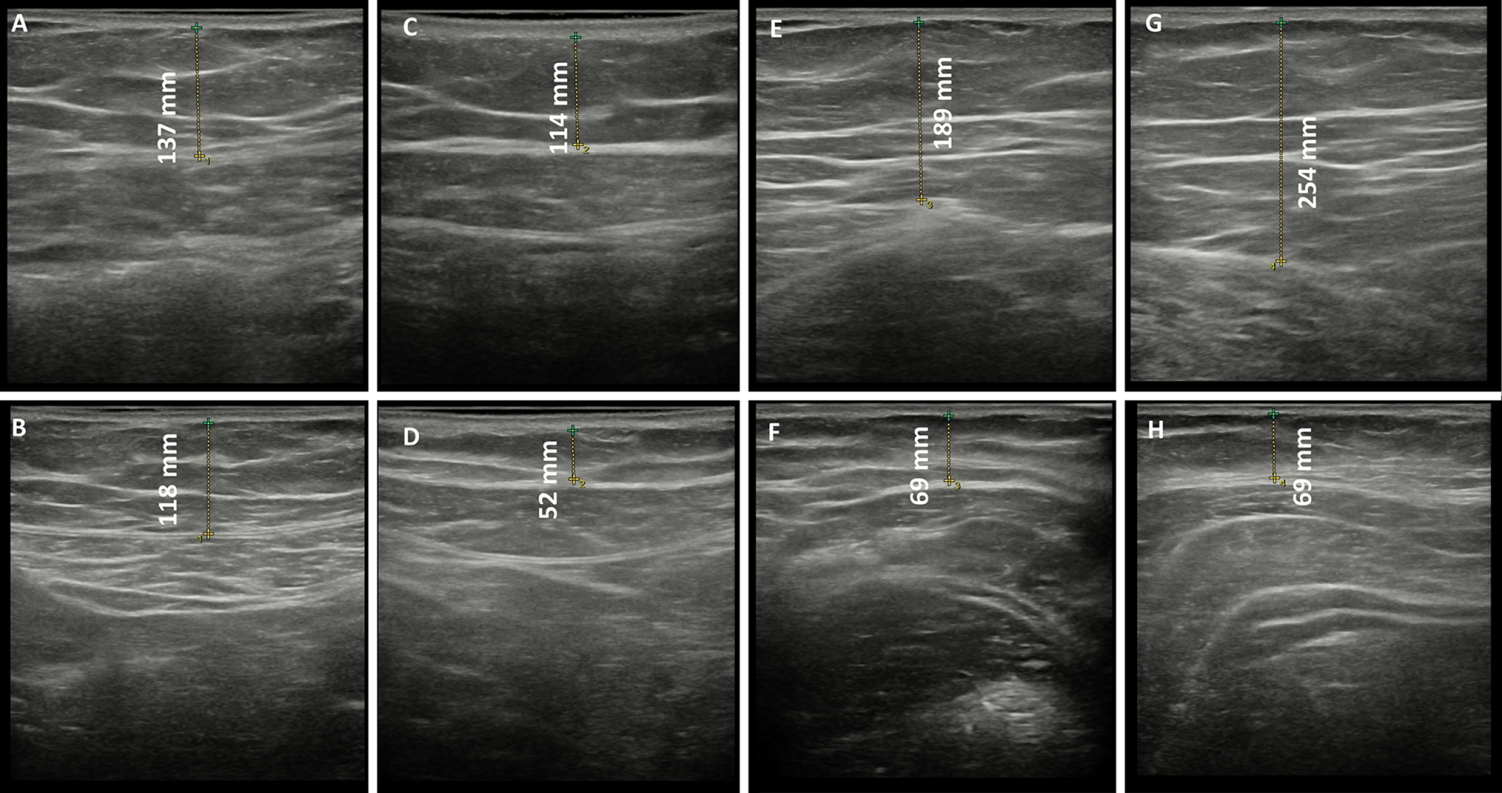
Ultrasound imaging of a 34-year-old woman who underwent four sessions in abdomen and hips with the new non-invasive body contouring device. **A** Infraumbilical thickness before treatment. **B** Infraumbilical thickness after treatment. **C** Supraumbilical thickness before treatment. **D** Supraumbilical thickness after treatment. **E** Right hip thickness before treatment. **F** Right hip thickness after treatment. **G** Left hip thickness before treatment. **H** Left hip thickness after treatment

Figures 1 and 2 show the fat thickness reduction, assessed by diagnostic ultrasound, in a 47-year-old woman who underwent treatment in abdomen and a 34-year-old woman who underwent treatment in abdomen and hips, respectively.

### Unadjusted Clinical Outcomes

Pretreatment fat mass was significantly reduced from 32.9% to 31.2% (Hodges-Lehmann median difference: − 1.7%; 95% CI − 3.4% to − 0.1%, *p* = 0.0006). There were no significant differences between pre- and posttreatment values in BMI, weight, and visceral fat (Table 3) yet significantly regarding fat mass % reduction and in thickness in the four areas evaluated.

**Table 3:** A comparison between pre- and post-treatment values.

	Pre-treatment	Post-treatment	Hodges-Lehmann median difference	*p*^a^
Weight, kg				0.1354
Median (IqR)	66.7 (59.0–75.3)	66.0 (56.6–74.3)	− 0.5 (− 1.2 to 0.3)	
BMI, kg/m^2^				0.1531
Meadin (IqR)	24.4 (22.1–27.1)	24.0 (22.0–27.2)	− 0.2 (− 0.4 to 0.1)	
Visceral fat				0.0625
Median (IqR)	5.0 (3.3–7.8)	5.0 (2.3–7.5)	− 0.5 (− 0.5 to 0.0)	
Fat mass, %				0.0006
Median (IqR)	32.9 (26.9–36.2)	31.2 (21.9–35.8)	− 1.7 (− 3.4 to − 0.1)	
FTIU, mm				0.0001
Median (IqR)	195.0 (154.3–275.0)	118.0 (6.0–155.0)	− 85.3 (− 107.5 to − 62.0)	
FTSU, mm				0.0001
Median (IqR)	174.0 (105.0–217.0)	81.0 (56.8–123.5)	− 70.3 (− 95.0 to − 48.5)	
FTRH^b^				0.0039
Median (IqR)	189.0 (112.8–289.3)	69.0 (51.0–115.3)	− 100.0 (− 140.5 to 49.5)	
FTLH^c^				0.0078
Median (IqR)	182.0 (90.5–264.5)	69.0 (63.5–139.0)	− 71.8 (− 132.5 to − 23.0)	

The Wilcoxon test was used to assess the p values between pre- and post-treatment values

*IqR* Interquartile range, *BMI* Body mass index, *FTIU* Fat thickness infraumbilical region, *FTSU* Fat thickness supraumbilical region, *FTRH* Fat thickness right hip, *FTLH* Fat thickness left hip, *N.A.* Not applicable

^a^Wilcoxon test

^b^Nine subjects

^c^Eight subjects

### Adjusted Clinical Outcomes

Once adjusted by age, weight, BMI, fat mass, visceral fat, and pre-treatment thickness our study did not find significant differences in any of the study variables between subjects who underwent treatment in abdomen and those who underwent treatment in abdomen and hip regions (Table 4).

**Table 4:** Evolution of fat thickness in the different regions.

	Abdomen		Abdomen and hips		Difference between treatment groups	
	Median (95% CI) difference from baseline	*p*^a^	Median (95% CI) difference from baseline	*p*^a^	Mean (95% CI)	*p*^b^
Weight, kg	− 0.6 (− 1.7 to 1.3)	0.4375	− 0.5 (− 1.9 to 0.6)	0.2500	0.4 (− 1.8 to 2.6)	0.6803
BMI, kg/m^2^	− 0.2 (− 0.6 to 0.4)	0.4375	− 0.2 (− 0.8 to 0.1)	0.2500	0.1 (− 0.6 to 0.8)	0.6497
Visceral fat	0.0 (− 1.0 to 0.0)	0.2353	− 0.5 (− 1.0 to 0.0)	0.1250	0.6 (− 0.5 to 1.7)	0.2624
Fat mass, %	− 1.6 (− 3.9 to 0.3)	0.0938	− 1.8 (− 6.0 to − 0.6)	0.0078	2.9 (− 1.5 to 7.3)	0.1664
FTIU, mm^c^	− 97.5 (− 127.0 to − 63.0)	0.0313	− 74.0 (− 119.5 to − 37.0)	0.0039	− 10.5 (− 53.5 to 32.5)	0.5821
FTSU, mm^d^	− 79.0 (− 172.0 to − 52.0)	0.0313	− 61.5 (− 98.5 to − 38.0)	0.0039	− 2.9 (− 24.8 to 18.9)	0.7602
FTRH	N.A.	N.A.	− 100.0 (− 140.5 to 49.5)	0.0039	N.A.	N.A.
FTLH	N.A.	N.A.	− 71.8 (− 132.5 to − 23.0)	0.0078	N.A.	N.A.

The Wilcoxon test was used to assess the intra-group statistical significance. The analysis of covariance (ANCOVA) was used for determining the *p* values between groups. “Treated area” as a factor and age, weight, BMI, visceral fat, and fat mass as covariates

*FTIU* Fat thickness infraumbilical region, *FTSU* Fat thickness supraumbilical region, *FTRH* Fat thickness right hip, *FTLH* Fat thickness left hip

^a^Wilcoxon test

^b^ANCOVA test with the Bonferroni correction

^c^Also adjusted by pretreatment infraumbilical thickness

^d^Also adjusted by pretreatment supraumbilical thickness

**Fig. 3:**
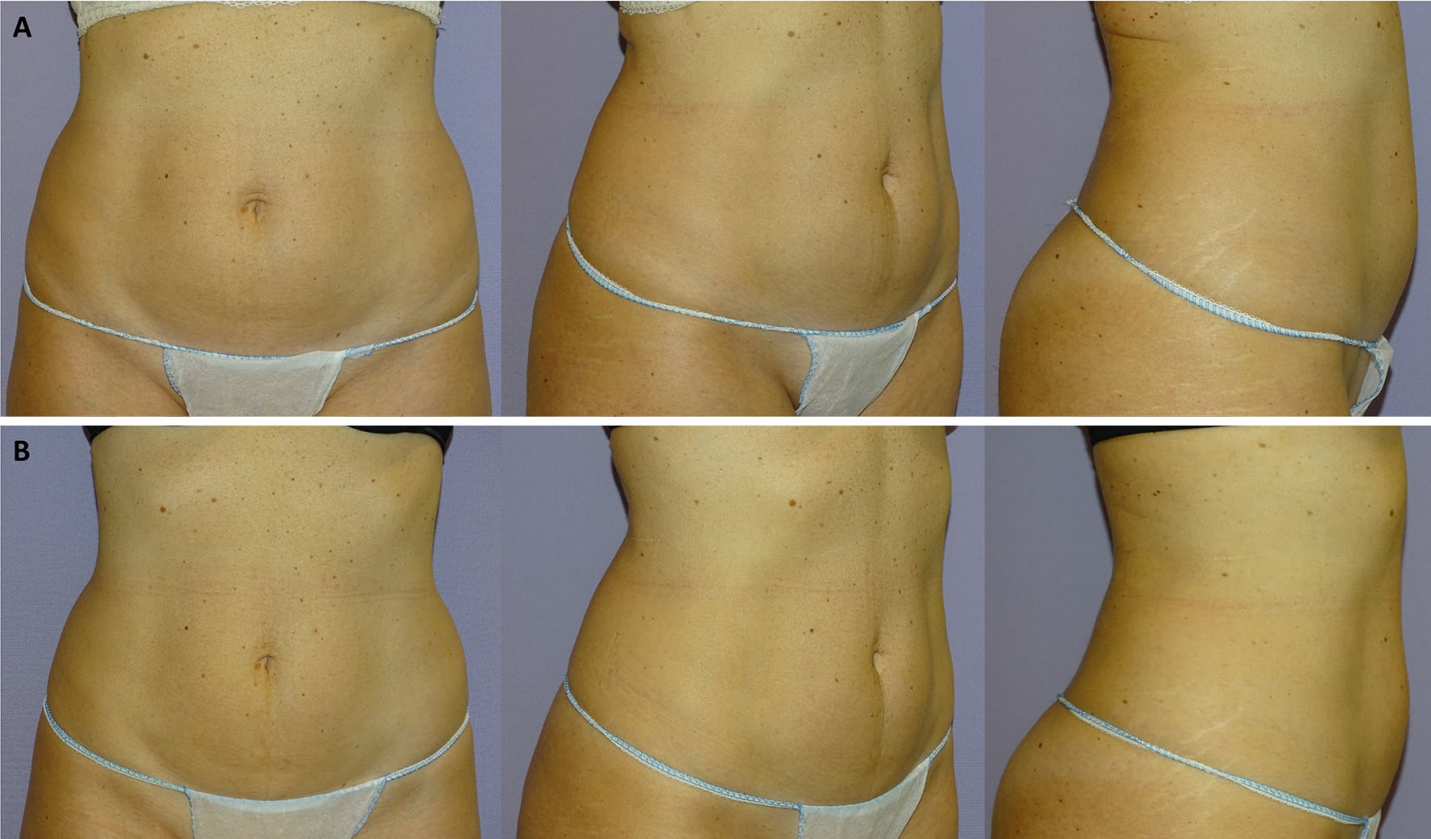
Frontal, right oblique, and lateral view of a 47-year-old woman before (**A**, respectively) and after (**B**, respectively) being treated (four sessions in abdomen) with the new non-invasive body contouring device Accent Prime X

**Fig. 4:**
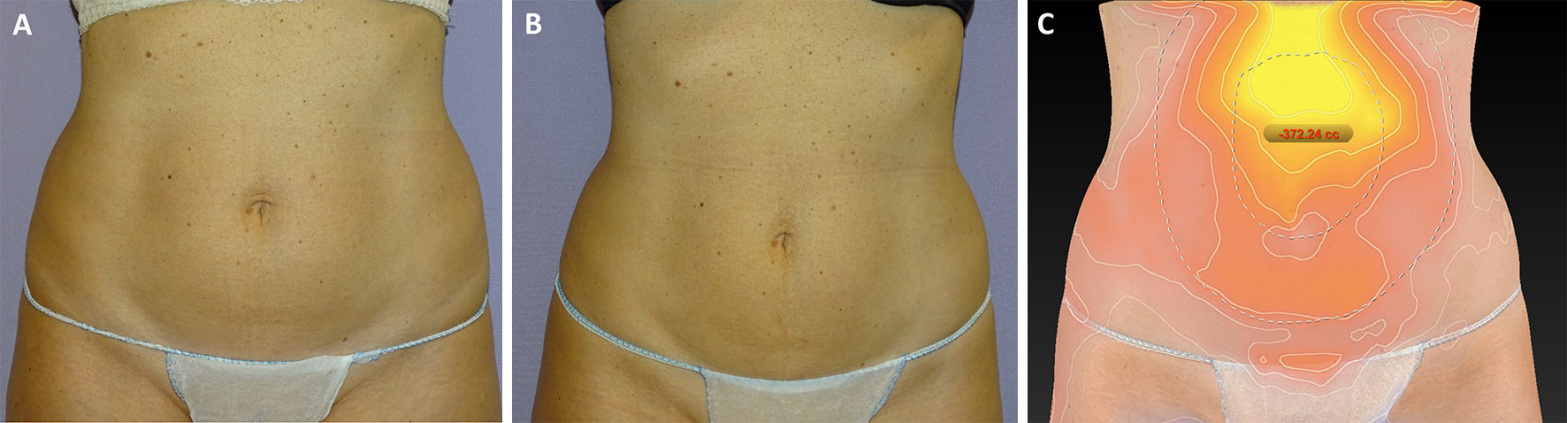
**A** comparison between the two-dimension photos with SonyDSC-HX400V (Sony Group Corporation Konan Minato-ku, Tokyo, 108-0075 Japan) and three dimension photographs with Canfield Vectra H2 (Canfield Scientific Inc; Parsippany-Troy Hills, NJ 07054, USA) of a 47-year-old woman (same patients Fig. 5) who underwent treatment in abdomen and hips. **A** Frontal view before treatment. **B** Frontal view after treatment. **C** The degree of volume between before and after was assessed by a color map. As compared to the pretreatment visit, the mean fat volume was reduced in 372.24 cc 3-months after treatment

**Fig. 5:**
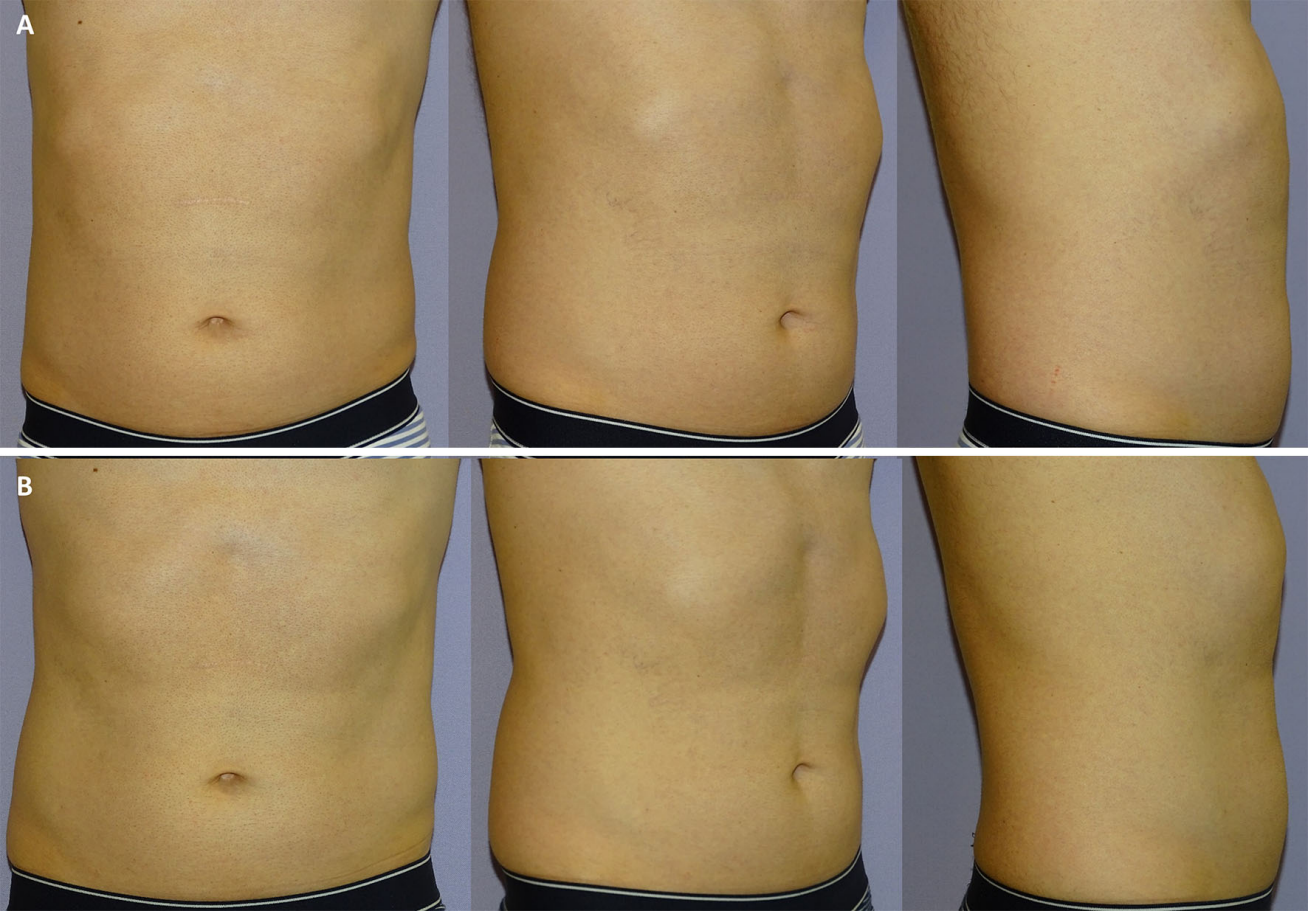
Frontal, right oblique, and lateral view of a 41-year-old man before (**A**, respectively) and after (**B**, respectively) being treated (four sessions in abdomen and hips) with the new non-invasive body contouring device Accent Prime X

**Fig. 6:**
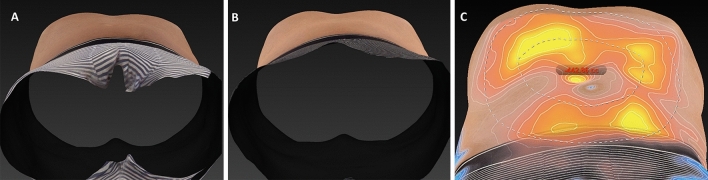
Three-dimension photographs with Canfield Vectra H2 (Canfield Scientific Inc; Parsippany-Troy Hills, NJ 07054, USA) of a 41-year-old man (same patients Fig. 6) who underwent treatment in abdomen and hips. **A** Sagittal view before treatment. **B** Sagittal view after treatment. **C** The degree of volume between before and after was assessed by a color map. As compared to the pretreatment visit, the mean fat volume was reduced in 442.86 cc 3-months after treatment

**Fig. 7:**
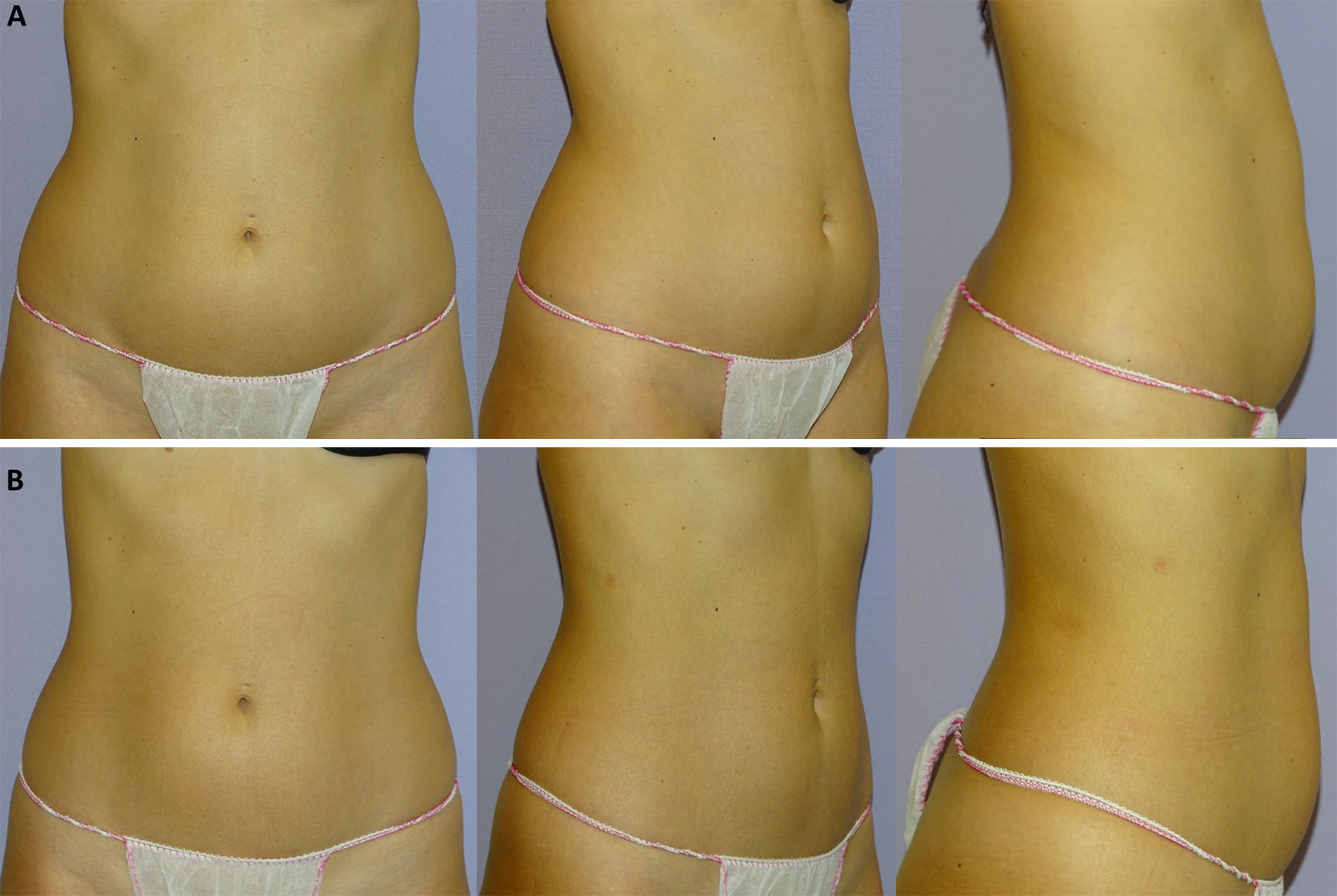
Frontal, right oblique, and lateral view of a 34-year-old woman before (**A**, respectively) and after (**B**, respectively) being treated (four sessions in abdomen and hips) with the new non-invasive body contouring device Accent Prime X

**Fig. 8:**
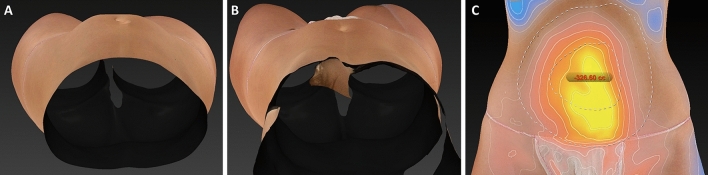
Three-dimension photographs with Canfield Vectra H2 (Canfield Scientific Inc; Parsippany-Troy Hills, NJ 07054, USA) of a 41-year-old man (same patients Fig. 6) who underwent treatment in abdomen and hips. **A** Sagittal view before treatment. **B** Sagittal view after treatment. **C** The degree of volume between before and after was assessed by a color map. As compared to the pretreatment visit, the mean fat volume was reduced in 326.60 cc 3-months after treatment

Clinical results, assessed by means of the 3D imaging system Canfield Vectra H2 or by 2D photographs, have shown a significant improvement in the aesthetic results (Figs. 3, 4, 5, 6, 7, 8).

The median (IqR) degree of patient satisfaction was 4.0 (4.0–5.0), with 13 (86.7%) patients being “Highly satisfied” or “Satisfied” with the treatment results, and only 2 (13.3%) ones who were “Neutral” with their esthetic results.

Regarding safety, the treatment was well tolerated. Adverse events were mild in severity and were successfully controlled without sequelae or need for medical or physical therapy. The most commonly reported adverse events were mild discomfort, immediately after treatment, which was successfully resolved without treatment; erythema (mild), which was successfully resolved without treatment within 48 h after treatment. No cases of burns or skin atrophy have been observed.

## Discussion

The results of the current evaluation suggest that the treatment with a non-invasive device that combines pulsed non-focus ultrasound and Unipolar radiofrequency provided a significant fat thickness reduction in abdomen and hips that lasts at least 3 months, with an excellent safety profile.

After adjusting by different covariates, our study did not find significant differences in fat reduction among patients who underwent treatment in abdomen and those who did it in abdomen and hips. Moreover, with the exception of the fat mass, which was significantly lower after treatment, during the follow-up period, there were no significant changes in weight, BMI or visceral fat, which suggested clearly that reduction in fat thickness was due to local treatment rather than behavioral causes.

Over the past several years, there has been a paradigm shift in the treatment of fat. The clear trend is toward noninvasive technologies over more traditional forms of fat removal such as liposuction [1–5, 7].

Non-invasive body contouring technologies can be classified by the energy source deployed, or by their effect on adipose tissue. In a more didactic way, these non-invasive therapies may be classified, according to their effect on the adipocyte, in short-term (metabolic size reduction) and long-term (permanent adipocyte death) effect devices [2–5].

Among the different non-invasive techniques used currently for body contouring, RF and US have become increasingly popular [3, 10, 15]. Adipose tissue is highly influenced by thermal changes. This fact, in combination with the development of new non-invasive ultrasound technologies, has led to the use of ultrasound as a strategy for thermal fat tissue destruction [13–16]. Although the effects of HIFU technology have been widely evaluated, the effects of non-focused ultrasound on the adipocyte have been less explored [13, 14].

From a theoretical point of view, the combination of RF with ultrasound targets the problem of localized fat accumulation from two fronts [3, 17, 18]. Moreover, a synergistic effect has been suggested when combining both techniques, amplifying effect of one therapy over the other [17].

Although it has not been fully elucidated, the reduction in adipose tissue after the treatment may be a result of lipolysis or mechanical disruption of subcutaneous adipocytes. Additionally, it has been hypothesized that the absorption of ultrasonic energy can lead to changes in the structure and cellular activity of adipocytes, which causes the liberation of lipids to the bloodstream and extracellular space immediately after the treatment [16, 19]. Fat clearance is performed by physiological pathways, namely lymphatic, venous, and immune systems. Triglycerides from the broken fat cells are released into the interstitial fluid and metabolized subsequently by Lipase enzyme into glycerol and free fatty acids [20]. Glycerol is phosphorylated and transported through the vascular system, while free fatty acids are metabolized and these fat metabolites are processed in the liver in the same manner as fat originating from digested food [20].

According to our results, it can be deduced that to treat the abdomen and hip in the same session is as effective and safety as that to treat only the abdomen. We have not observed synergies or antagonisms when we treat the two regions in the same session. Nevertheless, we did not observe any safety issues related to our technique.

It is extremely difficult to compare our results with the currently available scientific evidence. In fact, as far as we know, this is the first study evaluating the effectiveness of this non-invasive body contouring platform, incorporating its two technologies of ultrasound and RF.

The RF technology delivers a thermal stimulus to the skin and superficial adipose tissue causing a thickening of the dermis and enhancement of fat cell metabolism, resulting in a reduction in skin laxity and adipocyte volume [21, 22].

Different studies have shown a fat tissue reduction using RF, suggesting that RF is an effective and safe method for reducing localized fat, particularly in abdomen and thighs [23–25]. Additionally, this technique is less time-consuming than other ones, which may be an advantage.

Another key point of the current study was the assessment of patient’s satisfaction with the treatment. The median degree of satisfaction with the treatment results was high (4 out of 5), with 7 (46.7%) patients who were “satisfied” and 6 (40.0%) ones who were “highly satisfied” with the treatment results.

Regarding safety, the all the adverse effects were mild in severity and were successfully resolved without treatment. No severe adverse events, such as burns or skin atrophy, have been observed in the current study. RF and US are, in general terms, safe technologies for these procedures [3, 5]. Moreover, the absence of complications, such as abdominal pain, erythema, or burns, have been reported [26].

Among others, the main limitations of the current study are a small sample, the limited follow-up, and the lack of a control group.

## Conclusions

The results of this study suggested that the combine therapy with pulsed non-focus ultrasound and Unipolar radiofrequency was effective for reducing fat tissue thickness in abdomen and hips in patients with localized fat, while maintained a good safety profile. However, due to the limited sample size and the characteristics of our study, appropriate caution is therefore recommended when extending the results to other patients. New clinical trials including a larger sample size and a longer follow-up using this device will be necessary to confirm these findings.

## References

[1] Mazzoni D, Lin MJ, Dubin DP, Khorasani H (2019). Review of non-invasive body contouring devices for fat reduction, skin tightening and muscle definition. Australas J Dermatol.

[2] Lee NY, Robinson DM (2017). Noninvasive body contouring. Semin Cutan Med Surg.

[3] Rzepecki AK, Farberg AS, Hashim PW, Goldenberg G (2018). Update on noninvasive body contouring techniques. Cutis.

[4] Cox SE, Butterwick K (2020). Introduction to body contouring special issue. Dermatol Surg.

[5] Kennedy J, Verne S, Griffith R, Falto-Aizpurua L, Nouri K (2015). Non-invasive subcutaneous fat reduction: a review. J Eur Acad Dermatol Venereol.

[6] Shermak MA (2012). Body contouring. Plast Reconstr Surg.

[7] International Society of Aesthetica Plastic Surgery (Authors no listed). The international study on aesthetic/cosmetic procedures performed in 2019. Available at: https://www.isaps.org/wpcontent/uploads/2020/12/Global-Survey-2019.pdf. Accessed 1 Feb, 2022

[8] Narins RS, Tope WD, Pope K, Ross EV (2006). Overtreatment effects associated with a radiofrequency tissue-tightening device: rare, preventable, and correctable with subcision and autologous fat transfer. Dermatol Surg.

[9] Araújo AR, Soares VP, Silva FS, Moreira Tda S (2015). Radiofrequency for the treatment of skin laxity: mith or truth. An Bras Dermatol.

[10] Weiss RA (2013). Noninvasive radio frequency for skin tightening and body contouring. Semin Cutan Med Surg.

[11] Weiss R, Weiss M, Beasley K, Vrba J, Bernardy J (2013). Operator independent focused high frequency ISM band for fat reduction: porcine model. Lasers Surg Med.

[12] Chilukuri S, Mueller G (2016). “Hands-Free” noninvasive body contouring devices: review of effectiveness and patient satisfaction. J Drugs Dermatol.

[13] Garcia O, Schafer M (2013). The effects of nonfocused external ultrasound on tissue temperature and adipocyte morphology. Aesthet Surg J.

[14] Levi A, Amitai DB, Lapidoth M (2017). A novel transcutaneous, non-focused ultrasound energy delivering device is able to induce subcutaneous adipose tissue destruction in an animal model. Lasers Surg Med.

[15] Luo W, Zhou X, Gong X, Zheng M, Zhang J, Guo X (2007). Study of sequential histopathologic changes, apoptosis, and cell proliferation in rabbit livers after high-intensity focused ultrasound ablation. J Ultrasound Med.

[16] Minkis K, Alam M (2014). Ultrasound skin tightening. Dermatol Clin.

[17] Canela VC, Crivelaro CN, Ferla LZ, Canela VC, Crivelaro CN, Ferla LZ, Pelozo GM, Azevedo J, Liebano RE (2018). Synergistic effects of combined therapy: nonfocused ultrasound plus Aussie current for noninvasive body contouring. Clin Cosmet Investig Dermatol.

[18] Alizadeh Z, Halabchi F, Mazaheri R, Abolhasani M, Tabesh M (2016). Review of the mechanisms and effects of noninvasive body contouring devices on cellulite and subcutaneous fat. Int J Endocrinol Metab.

[19] Gonçalves WL, Graceli JB, Santos RL, Cicilini MA, Bissoli NS, Abreu GR (2009). Ultrasound lipoclasia on subcutaneous adipose tissue to produce acute hyperglycemia and enhance acute inflammatory response in healthy female rats. Dermatol Surg.

[20] El-Zayat SR, Sibaii H, El-Shamy KA (2019). Physiological process of fat loss. Bull Natl Res Cent.

[21] Zelickson BD, Kist D, Bernstein E, Brown DB, Ksenzenko S, Burns J (2004). Histological and ultrastructural evaluation of the effects of a radiofrequency-based nonablative dermal remodeling device: a pilot study. Arch Dermatol.

[22] Elsaie ML (2009). Cutaneous remodeling and photorejuvenation using radiofrequency devices. Indian J Dermatol.

[23] Manuskiatti W, Wachirakaphan C, Lektrakul N, Varothai S (2009). Circumference reduction and cellulite treatment with a TriPollar radiofrequency device: a pilot study. J Eur Acad Dermatol Venereol.

[24] Fritz K, Salavastru C (2017). Long-term follow-up on patients treated for abdominal fat using a selective contactless radiofrequency device. J Cosmet Dermatol.

[25] Boisnic S, Divaris M, Nelson AA, Gharavi NM, Lask GP (2014). A clinical and biological evaluation of a novel, noninvasive radiofrequency device for the long-term reduction of adipose tissue. Lasers Surg Med.

[26] Harth Y (2015). Painless, safe, and efficacious noninvasive skin tightening, body contouring, and cellulite reduction using multisource 3DEEP radiofrequency. J Cosmet Dermatol.

